# Role of Dendritic Cells in Viral Brain Infections

**DOI:** 10.3389/fimmu.2022.862053

**Published:** 2022-04-22

**Authors:** Orianne Constant, Ghizlane Maarifi, Fabien P. Blanchet, Philippe Van de Perre, Yannick Simonin, Sara Salinas

**Affiliations:** ^1^ Pathogenesis and Control of Chronic and Emerging Infections, Institut national de la santé et de la recherche médicale (INSERM), University of Montpellier, Etablissement Français du Sang, Montpellier, France; ^2^ Institut de Recherche en Infectiologie de Montpellier, Centre national de la recherche scientifique (CNRS), Université de Montpellier, Montpellier, France

**Keywords:** viral infection, dendritic cell, blood–brain barrier, neuroinflammation, neuroinfections, blood–cerebrospinal barrier

## Abstract

To gain access to the brain, a so-called immune-privileged organ due to its physical separation from the blood stream, pathogens and particularly viruses have been selected throughout evolution for their use of specific mechanisms. They can enter the central nervous system through direct infection of nerves or cerebral barriers or through cell-mediated transport. Indeed, peripheral lymphoid and myeloid immune cells can interact with the blood–brain and the blood–cerebrospinal fluid barriers and allow viral brain access using the “Trojan horse” mechanism. Among immune cells, at the frontier between innate and adaptive immune responses, dendritic cells (DCs) can be pathogen carriers, regulate or exacerbate antiviral responses and neuroinflammation, and therefore be involved in viral transmission and spread. In this review, we highlight an important contribution of DCs in the development and the consequences of viral brain infections.

## Introduction

The central nervous system (CNS) is often considered as an immune-privileged organ because it is separated from the blood by specific cellular barriers. Nonetheless, pathogens, in particular neurotropic viruses, have been selected throughout evolution for their ability to reach the brain (1). Human pathologies following CNS viral infections can be due to direct virus invasion and elicited toxicity or indirectly by mediators of neuroinflammation. For instance, some virus-associated neuronal diseases can be due to direct infection of neurons or to indirect effects triggered by CNS-supporting cells and inflammatory mediators, causing damages and dysfunctions (paralysis, cognitive deficits, ocular problems) (2, 3). Different clinical symptoms may appear depending of the site of infection and/or inflammation: meningitis for inflammation of leptomeningeal structures, myelitis for the inflammation of the spinal cord, and encephalitis for inflammation of parenchymal brain tissue (4), the latter being most common upon viral neuroinfections. Viral brain infections are generally limited by *in situ* innate immune response of the host and by the physical protection exerted by the brain barriers. These are complex multicellular structures forming an endothelium or an epithelium that separates the systemic circulation from the CNS. Depending on their location, the brain barriers exert different functions but they mostly control diffusion between CNS and blood and allow a precise regulation of CNS metabolism and immunity.

Importantly, immune cells have an ambivalent role during viral brain infections, as they are actors of the antiviral response but can also be viral carriers into the CNS and therefore participate in virus transmission, neuroinflammation, and associated deleterious effects. Moreover, because of the presence of viruses or inflammatory mediators in the brain, the release of cytokines and chemokines will further allow transmigration of immune cells such as monocytes, T lymphocytes, natural killer cells (NK), and dendritic cells (DCs) (5–7).

Immunosurveillance is provided by specific resident and incoming immune cell subsets. Their distribution varies depending on the nature of the epithelium and the inflammatory state of the tissue (8, 9). Among these immune cells, DCs are the very first responders following viral infection, as they are at the frontier between innate and adaptive immunity and acting as sentinels of the immune system, notably in the site of potential infection, including in the CNS. In this review, we try to shed some light on the pathogenesis of viral brain infections with a particular interest in the interactions with brain barriers and a focus on the role of DCs during viral brain invasion.

## Mechanisms of Viral Brain Access

Different viral CNS access mechanisms have been characterized, involving a neuronal-mediated spread mechanism (through axonal transport) and a hematogenous-mediated viral entry route (through interaction with brain barriers) (**Table 1**). Notably, entry mechanisms and viral tropism may determine neurological symptoms and clinical outcome (42, 43).

**Table 1:** Different CNS mechanism access and symptoms for some neurotropic viruses.

Viruses	CNS access	Main cells targeted	Symptoms	References
Rabies virus	Axonal transport (peripheral nerve and olfactory bulb)	Neurons	Encephalitis	(10–13)
Herpes simplex virus	Axonal transport (olfactory bulb)	Glial cells (astrocytes) Neurons	Encephalitis, persisting latent infection	(14)
Poliovirus	Axonal transport (peripheral nerve and olfactory bulb) Entry *via* brain barrier	Neurons	Paralytic poliomyelitis, encephalitis, acute flaccid paralysis	(15, 16)
St. Louis encephalitis virus	Axonal transport (peripheral nerve and olfactory bulb) Entry *via* brain barrier	Neurons Glial cells (astrocytes)	Meningitis, encephalitis, coma, agitations, confusion, tremors	(17)
West Nile virus	Axonal transport (peripheral nerve and olfactory bulb) Entry *via* brain barrier Cell-mediated (leukocytes neutrophils)	Neurons Endothelial cells Astrocytes	Encephalitis, cognitive dysfunction, flaccid paralysis, ocular manifestations, muscle weakness	(18–22)
SARS-CoV-2	Axonal transport (olfactory bulb)	Neurons?	Seizures, encephalitis, loss of consciousness, anosmia, ageusia, Guillain–Barré syndrome, ischemic stroke	(23, 24)
Measles	Axonal transport (peripheral nerve and olfactory bulb) Cell-mediated Entry *via* brain barrier	Neurons BBB endothelial cells	Encephalitis, encephalomyelitis, subacute sclerosing, panencephalitis	(25, 26)
HTLV-1	Entry *via* brain barrier	Neurons	HTLV-associated myelopathy/tropical spastic paraparesis (HAM/TSP)	(27)
Zika	Axonal transport (peripheral nerve and olfactory bulb) Cell-mediated (leukocytes) Entry *via* brain barrier	Neurons BBB cells Glial cells	Guillain–Barré syndrome, congenital Zika syndrome, meningoencephalitis	(28–30)
Chikungunya virus	Entry *via* brain barrier	Neurons Glial cells (astrocytes)	Myalgia, arthralgia, encephalopathy, hemorrhagic fever, meningoencephalitis, myelitis, Guillain–Barré syndrome	(31)
Echovirus-30	Entry *via* brain barrier (infection choroid plexus)		Meningitis, encephalitis, flaccid paralysis, myocarditis	(32)
JC virus	Entry *via* brain barrier (infection choroid plexus) Cell-mediated (B cells)	Oligodendrocytes Microglial cells	Encephalitis, meningoencephalitis, multifocal leukoencephalopathy	(33, 34)
HIV-1 (and SIV)	Cell-mediated (CD4+ T cells, monocytes, DCs) Entry *via* brain barriers	Macrophages Microglia	HIV-1-associated dementia (HAD), cognitive and motor disorders, HIV-1-associated neurocognitive disorders (HAND)	(35–37)
Coxsackievirus	Cell-mediated (myeloid cells) Entry *via* brain barrier (infection choroid plexus)	Neurons	Encephalomyelitis, meningitis	(38)
Toscana virus	Cell-mediated Entry *via* brain barriers	Brain endothelial cells Neurons?	Kernig sign, nuchal rigidity, photophobia, consciousness troubles, tremors, nystagmus, paresis, meningitis, meningoencephalitis, encephalitis	(39)
Varicella zoster virus	Cell-mediated (DCs and T cells) Entry *via* brain barriers	Nerve cells	Postherpetic neuralgia, congenital varicella syndrome	(40)
Nipah virus	Cell-mediated Axonal transport Entry *via* brain barriers	Brain endothelial cells Neurons	Encephalitis, vasculitis, parenchymal necrosis drowsiness, headache, disorientation or confusion, reduced consciousness	(41)

BBB, blood-brain barrier; HIV, human immunodeficiency virus; HTLV, human T-cell lymphotrophic virus; SARS-CoV-2, severe acute respiratory syndrome coronavirus 2; SIV, simian immunodeficiency virus.

### Axonal Transport

**Figure 1:**
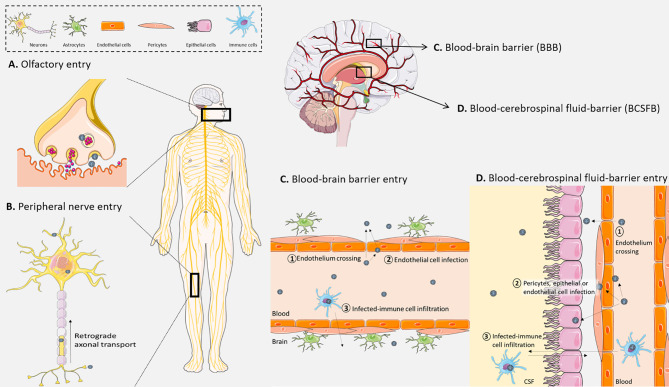
Different viral modes of access to the central nervous system (CNS). **(A)** Viruses can be up taken by nerve terminals at the olfactory bulb to gain access to the CNS. **(B)** They also can infect peripheral neurons and use long-range retrograde axonal transport. **(C)** At the blood–brain barrier (BBB), (1) viruses can directly cross the endothelium (by paracellular or transcellular ways), (2) they can infect and replicate in brain endothelial cells and be released in the CNS, (3) and finally they can pass through the BBB by infecting immune cells that cross the endothelium through the “Trojan horse” mechanism. **(D)** At the blood–cerebrospinal fluid barrier, (1) viruses can directly cross the endothelium, (2) they can also infect, replicate, and be released from endothelial cells, pericytes, or epithelial cells of the choroid plexus, (3) and finally infected immune cells can also cross this barrier and deliver viruses in the CNS. Created with SMART Servier Medical Art.

Neurons represent significant entry gates to the CNS for numerous pathogens (43). Indeed, cranial nerves from the olfactory system (**Figure 1A** **)** or peripheral nerves (**Figure 1B**) can be used for axonal transport by viruses such as rabies virus, herpes simplex virus (HSV), poliovirus, St. Louis encephalitis virus, West Nile virus (WNV), or severe acute respiratory syndrome coronavirus 2 (SARS-CoV-2) (10–12, 14–16, 18, 19, 23, 24). Viruses use also the host cell transport machinery, such as transport mediated by kinesins along microtubules in the anterograde direction or the interaction of dynactin and dynein with microtubules in the retrograde transport (44–46) (**Figure 1B**) and trans-synaptic trafficking that will enhance brain invasion as it was described for WNV (47) and Measles virus (25) (**Figure 1A** **)**. This allows a direct access to the CNS while escaping from host immune response (17, 48, 49). For instance, some viruses can access the CNS *via* peripheral uptake because of limited host defense that did not control peripheral infection (1).

### Entry Through the Blood–Brain Barrier

The blood–brain barrier (BBB), located between neuronal capillaries and the CNS, is constituted by pericytes and astrocytes closely interacting with a monolayer of endothelial cells to control exchanges with the blood through the high expression of tight junction proteins **(** **Figure 1C** **)** (50–52). This structure is found in cerebral blood vessels and is essential for the transport of lipid-soluble molecules or gaseous and liquid components by passive diffusion, while the transport of large and polar molecules is reduced (53). The BBB also has active transport mechanisms that regulate CNS homeostasis while avoiding neuroinvasion by leukocytes or pathogens (54). However, some molecules and pathogens can be transported in endocytic vesicles across the endothelial cells and pericytes, and transferred into the CNS, such as cells and pathogens upon secretion of chemoattractant molecules (28, 55).

Cell-free viruses, when in sufficient amount in the blood, can reach the CNS during the primary viremia (**Figure 1C**). Thereupon, endothelial cells can be susceptible and permissive to direct viral infection and replication, leading to brain invasion by basolateral viral particle release. Measles virus, for example, can productively infect BBB endothelial cells, allowing the release of viral particles and CNS invasion (26). Other studies on the interaction between human brain endothelial cells and flaviviruses have demonstrated CNS invasion by direct infection of these cells (**Figure 1C**) (28, 56). Moreover, the presence of viral particles such as WNV in the blood can also lead to BBB dysregulation with a decrease of tight junction protein expression, mostly zona occludens-1 (ZO-1) and claudin-5, after activation of Rho GTPases following the recognition of pathogen-associated molecular patterns (PAMPs) by pattern recognition receptors (PRRs). For instance, human T-cell lymphotrophic virus type 1 (HTLV-1) is also able to productively infect human brain endothelial cells, leading to a dysregulation of tight junction protein expression and subsequent transcellular virus spread into the CNS (27, 57).

### Viral Interaction With Meninges and the Blood–Cerebrospinal Fluid Barrier

The blood–cerebrospinal fluid (CSF) barrier (BCSFB) limits the passage between the blood and the CSF produced by the choroid plexus (51) (**Figure 1D** **).** Located in the ventricular system of the brain, the choroid plexus complexed with endothelial cells and CSF form the BCSFB. The stroma, at the center of the choroid plexus, is composed of fibroblasts, immune cells, connective tissue, and blood microvessels (58). Similarly to the BBB, this barrier is an important interface between the peripheral circulation and the CNS. Indeed, the specialized cuboidal epithelial cells form a layer with a high expression of tight junctions to separate the blood from the CSF that they produce (51, 59). Some viruses display important choroid plexus tropism such as chikungunya virus, echovirus-30, JC virus (JCV), or Zika virus (ZIKV) (31–33, 60). Neuroinvasion and/or spreading may also involve meninges and the CSF. Of note, the lymphatic transport system (glymphatic system and meningeal lymphatic vessels) is also a key actor in the regulation of CNS homeostasis due to its functions in immune monitoring and metabolite draining among others (61). It is also involved in pathological mechanisms, including brain infections (62). Because the lymphatic transport system is involved in CSF drainage (or outflow), it can also facilitate cell and viral CNS access. Indeed, evidence of close interaction between CSF and lymphatic vessels, notably for CSF drainage from subarachnoid space (63, 64), can support cell-mediated and viral particle circulation in the CNS.

### The Trojan Horse Mechanism

Importantly, an exacerbated primary inflammatory response that alters brain barrier permeability can also facilitate transfer through circulating infected immune cells (**Figure 1C**). The transfer of pathogens to the CNS through infected immune cells is called “Trojan horse” mechanism and is increasingly studied, as several viruses are now described to use this pathway to invade the CNS (65). CNS infection by human immunodeficiency virus (HIV)-1 can presumably occur in a cell-free manner (66), but virus transport across the brain barriers can be mediated by HIV-1-infected immune cells as monocytes or CD4^+^ T cells (35, 36, 66). HIV-1-infected CD14^+^/CD16^+^ monocytes were also reported to efficiently cross the BBB, thus placing the “Trojan horse” strategy as a main route of CNS infection (4, 67, 68). In non-human primates, it was also demonstrated in the role of simian immunodeficiency virus (SIV)-infected monocytes in neuroinvasion (37). Similarly, JCV can infect B cells, which transmigrate across the BBB (34). By its location at the interface of the blood, CSF, and brain, the choroid plexus is an important regulator of immune cell traffic and is also a target during infections and immune cell infiltration supporting neuroinflammation (59). For example, myeloid cells infected by coxsackievirus are suspected of being disseminators of the virus from the blood within the CNS *via* the choroid plexus (38).

**Figure 2:**
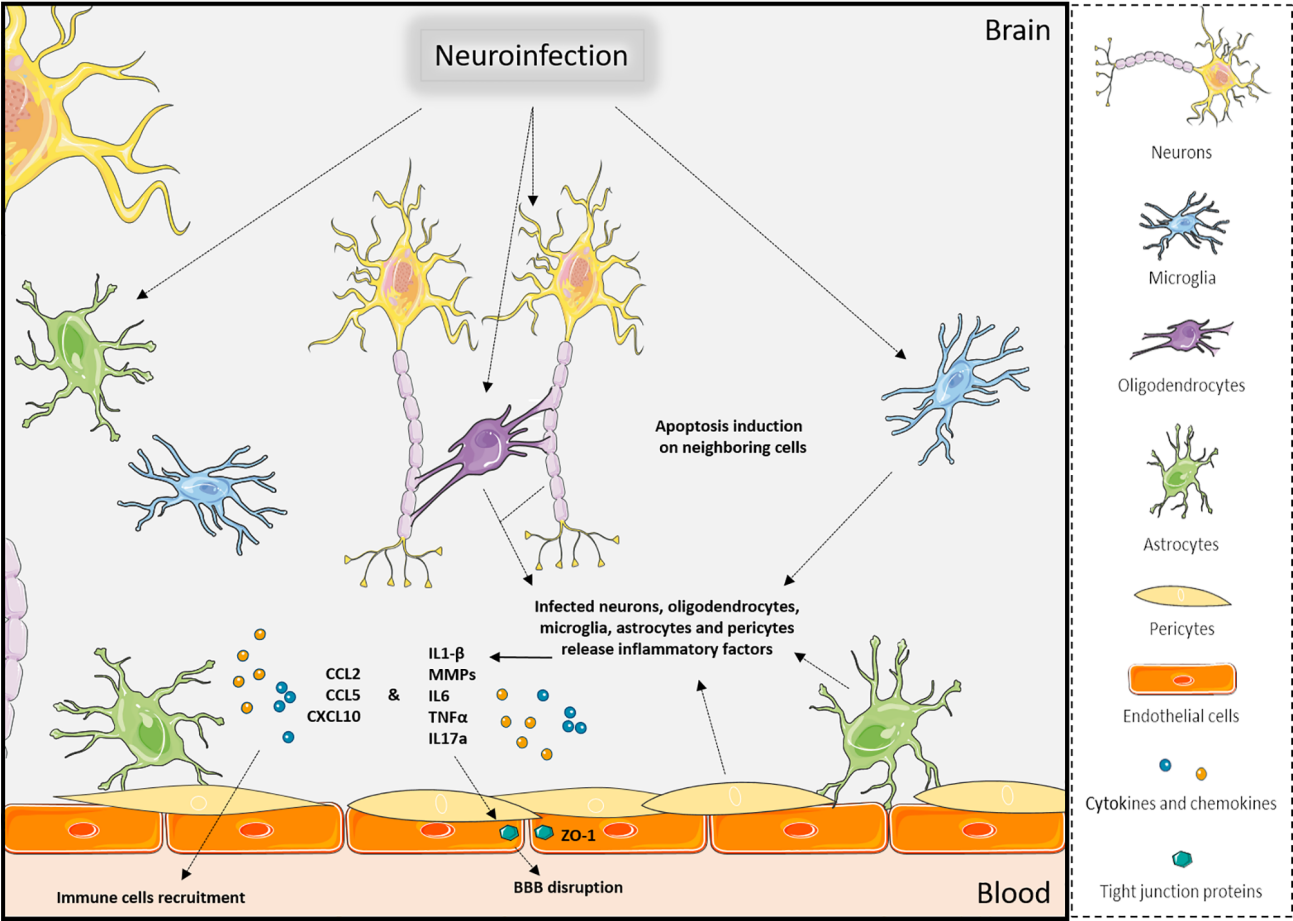
Effects of neuroinfections in the CNS. Viral brain infections will trigger multiple molecular and cellular mechanisms in the various CNS cell types that can lead to apoptosis of neuronal cells, release of pro-inflammatory factors, disruption of brain barriers, and immune cell recruitment, which ultimately will exacerbate neuroinflammation. Created with SMART Servier Medical Art.

Nonetheless, it seems that viral access through brain barriers and direct neuronal access to the CNS are not mutually exclusive and can occur concomitantly for instance during infection by rabies virus and WNV (1, 13, 17, 47, 69) (**Table 1**). Mediators released during infection can interact with and modulate the BBB permeability as vasogenic and growth factors, cytokines, and chemokines (70, 71). These examples illustrate how infections and pro-inflammatory factors lead to an increase in brain barrier permeability and an enhanced access across brain barriers, promoting the development of neuroinflammation (71–73) (**Figure 2**).

Upon cytokines, chemokines, and cellular adhesion molecule upregulation, different classes of immune cells can be recruited at brain barriers. Their transmigration into the CNS helps the antiviral response and control neuroinflammation or facilitate viral entry (**Figure 2**). In healthy context, macrophages and DCs can be found in the perivascular space at the BBB and choroid plexus, which ensure immunosurveillance. During pathological conditions, these cells and other antigen-presenting cells (APC) can recruit effector immune cells in these perivascular spaces (74). For example, in the context of SIV infection, SIV-infected immune cells are found in the perivascular space (37). This compartment should not be neglected during neuroinflammation studies, as immune cell recruitment at the BBB starts with a transmigration in the perivascular space before migration across glia limitans (75, 76).

## A Focus on Dendritic Cells

Immunosurveillance is provided by specific resident and incoming immune cell subsets. Their distribution varies depending on the nature and the inflammatory state of the tissue (8, 9). Among these immune cells, DCs are the very first responders following viral infection, as they are at the frontier between innate and adaptive immunity and acting as sentinels of the immune system, including in the CNS.

DCs include different subsets with specific cellular and immunological properties: the myeloid/conventional DCs (DCs), the Langerhans cells (LCs) that are the unique DC subset located in mucosal stratified (intestine and oral mucosa) and pseudostratified epithelium (example: lung) as well as skin epidermis (8, 77), and the plasmacytoid DCs (pDCs), which are unconventional DCs characterized by their ability to produce large amounts of type I interferon (IFN-I) in response to viral pathogens (78, 79) (**Table 2**).

**Table 2:** Phenotypic and functional markers of human blood and tissue dendritic cell subsets.

	Plasmacytoid DCs (pDCs)	Myeloid DC subtypes				
		Myeloid DCs	Langerhans cells (LCs)	Conventional DCs		Monocyte- derived DCs (MoDCs)
				cDC1	cDC2	
**Localization**	Blood	Blood	Epidermis and other tissues	Dermis and other tissues		*In vitro*
**Phenotype**	CD11c^–^CD1a^+^ CD1c^–^CD123^high^ BDCA4^+^ BDCA2^+^	CD11c^+^CD1a^+^ CD1c^+^CD123^low^ BDCA1+	CD11c+CD1a+ CD207+	CD11c^low^ CD68+ CD11b- CLEC9A^+^ XCR1^+^	CD11c+ CD11b+ SIRPa	CD11c+CD1a+ CD1c+CD123^low^
**TLR (toll like receptor) expression**	TLR1, TLR6, TLR7, TLR9 and TLR10	TLR1,TLR2, TLR3, TLR4, TLR5, TLR6, TLR8 and TLR10	TLR1, TLR2, TLR3, TLR6, TLR7 and TLR8	TLR1, TLR2, TLR3, TLR4, TLR5, TLR6, TLR7 and TLR8		TLR1, TLR2, TLR3, TLR4, TLR5, TLR6, TLR8 and TLR10
**C-type lectin expression**	BDCA2 and DCIR	DCIR, DC-SIGN and MR	Langerin/CD207	DC-SIGN and MR		DCIR, DC-SIGN and MR

BDCA2, blood DC antigen 2 (also known as CLEC4C); DCIR, DC immunoreceptor (also known as CLEC4A); MR, mannose receptor; SIRPa, signal regulatory protein a. BDCA2, blood DC antigen 2 (also known as CLEC4C); CD, cluster of differentiation; cDC, conventional dendritic cells; CLEC, C-type lectin domain containing; DC-SIGN, dendritic cell-specific intercellular adhesion molecule-3-Grabbing Non-integrin; DCIR, DC immunoreceptor (also known as CLEC4A); MR, mannose receptor; pDC, plasmacytoid dendritic cells; SIRPa, signal regulatory protein a; TLR, Toll-like receptor; XCR, X-C motif chemokine receptor.

DC subsets originate from bone marrow CD34^+^ hematopoietic stem cells giving rise to common myeloid/lymphoid progenitors (CMLPs), which then differentiate toward common myeloid progenitor (CMP) or common lymphoid progenitor (CLP). Under specific environmental cytokine conditions, concomitantly with the expression of defined transcription factors, CMPs separate from the monocyte/macrophage axis and generate common DC progenitors (CDPs), which will give rise to pDCs and conventional DC subsets (8, 80). Various DC precursors (Pre-DCs) with selective functional features are characterized by the unique expression of AXL and SIGLEC6 along with myeloid and plasmacytoid markers like CD11c and CD123 (81, 82). However, this was recently challenged, since DC (Axl^+^ Siglec 6^+^), named AS DC, was described in 2017 as a potential new functional DC subset instead of being an exclusive pre-DC progenitor (81). Nevertheless, DCs can be divided into three major DC subsets, pDC, conventional DC1 (cDC1), and conventional DC2 (cDC2), but other cell phenotypes can arise from those subsets particularly when considering tissue-resident DCs and inflammatory status (8, 80). Interestingly, LCs, which represent a DC subset exclusively present in the epidermis and upper mucosal layers, were shown to be functionally related to DCs but ontogenically closer to macrophages due to their reported embryonic origin and self-renewing capacities (83, 84). Indeed, the ontogeny of LCs has always been subject to intense debate, and new transcriptomic data suggest that LCs are rather closely related to tissue-resident macrophages. Nevertheless, LCs were recently reported to share ontogeny and transcriptional features with both macrophages and DCs (85). From a functional and phenotypic point of view, LCs have strong similarities with DCs (86). For example, LCs have the ability to capture pathogens and possess migratory abilities to initiate T-cell responses in lymph nodes. Moreover, LCs can be generated *ex vivo* from hematopoietic precursors in response to cytokines and cell membrane-associated ligands. Thus, LCs were found to share both macrophage and DC characteristics (85, 87), as particularly evidenced upon skin damage and inflammation or infection processes *in vivo*. All the current debates based on the different reported cell or animal models emphasize the importance to consider functional lineage plasticity besides ontogenetic data. Also, as LCs, other DC subsets were reported to develop under certain conditions like monocyte-derived DC (MoDC), arising in inflamed tissues and reminiscent of reported inflammatory DC subsets (88, 89). Importantly, the myeloid DC lineages (cDC1 and cDC2) are clearly distinguished from MoDCs, which are usually derived from monocytes predominantly under inflammatory conditions *in vivo* and *in vitro* (**Table 2**) (90), although the identification of new DC subsets evolves with the recent identification of new human anogenital MoDCs present in non-inflamed tissues that take up HIV and transmit virus to CD4 T cells (91).

DCs are highly specialized professional APC populations through the expression of major histocompatibility complex (MHC) class II and costimulatory molecules that lead to naive T-cell stimulation (91–94). They have the capacity to capture antigen in the periphery and in the subepithelium to migrate to proximal lymph nodes where they prime naive T cells and engage the adaptive immune response. These cells express various pathogen sensors, including many specific PRRs and toll like receptors (TLRs), allowing major secretion of type I IFN that induces IFN-stimulated genes (ISGs) used to counteract infection (8, 95–97). However, their capacity to capture pathogens is mainly due to their expression of lectin and lectin-like receptors such the sialic acid-binding immunoglobulin (Ig)-like lectins (Siglecs) family (98) and especially C-type lectins receptors (CLRs). CLRs bind carbohydrate structures associated with viruses, fungi, or bacteria expressed by LCs, Dendritic cell-Specific Intercellular adhesion molecule-3-Grabbing Non-integrin (DC-SIGN) (CD209) and the CLR DC-associated C-type lectin-2 (Dectin-2/CLEC6A) expressed by DCs and macrophage, and galactose type C-type lectin (MGL, CD301) expressed by both DCs and macrophages (but not LCs), respectively (99, 100). Although CLRs have established antiviral functions, many viruses are capable of hijacking these receptors to their advantage. In particular, langerin escape or its hijacking by a virus seems to be much rarer in contrast to DC-SIGN that has been shown to bind the gp120 glycoprotein of HIV-1 and promotes efficient trans-infection of CD4^+^ T cells. DC-SIGN can also be used to infect certain DCs with many viruses, such as cytomegalovirus or Ebola virus but also several flaviviruses, including dengue virus and WNV (101). Thus, some DC subtypes may constitute the point of access of viruses, allowing their subsequent transmission and propagation through the body including the CNS. Although the contribution of different DCs is not clearly established, they may also represent a “Trojan horse” because they can migrate efficiently to the brain.

## Dendritic Cells in Neuroinflammation and Brain Diseases

DCs are naturally present within the brain where they act as sentinels under steady-state conditions and during brain disease and neuroinflammation (102). They have been shown to efficiently interact with brain barriers in order to access the CNS.

**Figure 3:**
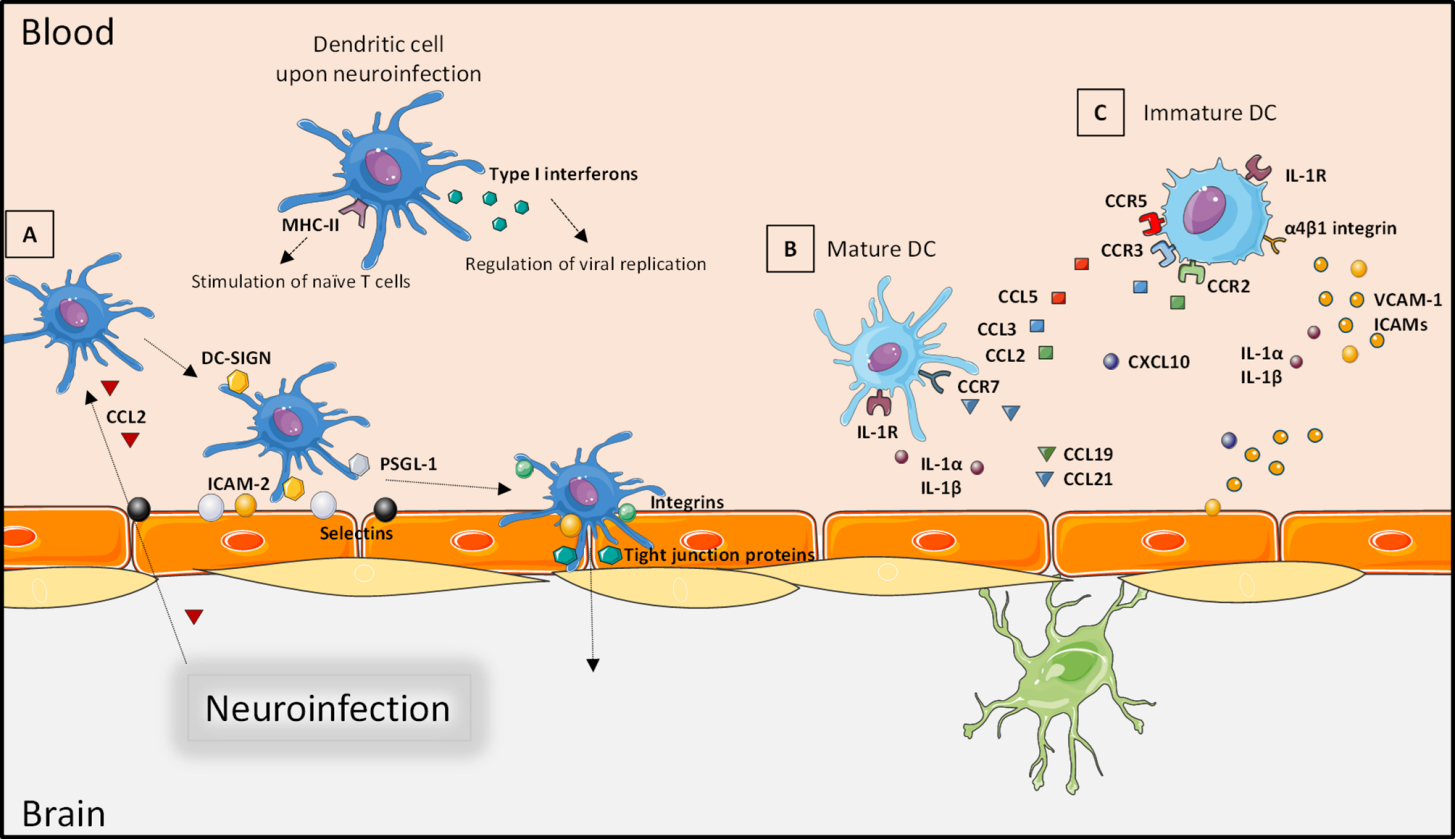
Interaction of dendritic cells with the blood–brain barrier. Through the neuroinfection and the release of pro-inflammatory factors, DCs can produce type I interferon to regulate viral replication. Infected DCs can also act as antigen-presenting cells and stimulate T cells. **(A)** DCs are chemoattracted to brain barriers by circulating chemokines (such as CCL2); a firm contact is established by interaction of cellular adhesion molecules expressed by endothelial cells that facilitate rolling and adhesion. Transmigration occurs as an interaction of cellular adhesion molecules and integrins. **(B)** The recruitment of mature DCs is facilitated by expression of CCR7 and IL-1R that increases attraction by secreted CCL19, CCL21 or IL-1α and IL-1β, respectively. **(C)** Immature DCs can also be attracted by interaction of IL-1α or IL-1β with IL-1R, but their expression of CCR2, CCR3, and CCR5 increases the interactions with CCL2, CCL3, and CCL5. Created with SMART Servier Medical Art.

During the leukocyte recruitment at the BBB, immune cells including DCs interact with various actors. First, cytokines and chemokines, released during CNS inflammation such as CCL2, can chemoattract circulating DCs at brain barriers. Then, contact between the upregulated cellular adhesion molecules (CAMs) P-selectin, E-selectin, vascular cell adhesion molecule-1 (VCAM-1), and intercellular adhesion molecule-2 (ICAM-2) on endothelial cells with P-selectin glycoprotein ligand-1 (PSGL-1) and DC-SIGN on DCs allows rolling and adhesion on the BBB apical surface (103). These steps are followed by potential transmigration, facilitated by interaction of DC-SIGN with CAMs or tight junction proteins. Interestingly, DCs can also express tight junction proteins to facilitate this step without a deleterious effect on the BBB integrity (103) (**Figure 3A**). Importantly, neuroinfiltration may depend on the DC state. Indeed, during activation and arrest steps, migration of mature DCs across the BBB can be enhanced by interaction of CCR7 with CCL19 and CCL21 (**Figure 3B**), whereas immature DCs can also be attracted by CCR2, CCR3, and CCR5, which interact with CCL2, CCL3, and CCL5, respectively (**Figure 3C**) (6, 104). Interestingly, immature DCs have better capacity to cross the BBB than mature DCs in part because of the different expressions of specific traffic signals at their surface (105, 106). *In vitro*, the transmigration of granulocyte-macrophage colony-stimulating factor (GM-CSF)-induced DCs across the BBB was shown to be increased in the presence of CCL3. In addition, GM-CSF-matured DCs secrete metalloproteinases, allowing transmigration across the BBB with redistribution of tight junction molecules (107). Regarding the BCSFB, molecular mechanisms of DC interaction are less well-defined (108, 109). As in the blood compartment, DCs are mainly recruited in the CSF by chemoattraction through the release of CCL2, CCL3, CCL4, and CXCL12, the latter of which being critical during this recruitment step (110). Following increased concentration of DCs in the CSF, they can interact by the expression of CAMs on their surface and on the surface of choroid plexus epithelial cells (5). All these interactions support BCSFB immunosurveillance functions with DC’s high APC role and recruitment in brain parenchyma.

Brain DCs represent 1% of immune cells found in the brain and are sparsely found in the CSF (less than 1% of CSF cells) (5, 111, 112). Macrophages and DCs are found naturally in non-pathological conditions in the choroid plexus stroma (113), in sites of plasticity and neurogenesis, and where the BBB is absent to provide immunosurveillance (114). Through their innate properties, brain DCs can activate encephalitogenic T cells (self-antigen-reactive T cells) or release pro-inflammatory cytokines to the detriment of the CNS homeostasis; their loss leads to dysregulation of immune tolerance and excessive inflammatory response (115). Consequently, in a context of adaptive immune response, DCs are important potential inducers of primary T cells (113). For example, they are among the APCs, present in dural sinuses, one compound of the meninges, and they allow the presentation of antigen from CSF to T cells in cervical lymph nodes (115, 116).

During neuroinflammation, the number of DCs increases in the CNS and particularly in the CSF where they can more easily reach lymph nodes to activate the immune response. They participate indirectly to exacerbate neuroinflammation (74, 110). For example, they are involved in autoimmune diseases as in the murine model of multiple sclerosis (MS) named experimental autoimmune encephalomyelitis (EAE) (117). MS is a chronic CNS inflammatory disease, often studied with the EAE mouse model, based on the reactivity of T cells to endogenous myelin epitopes (encephalitogenic T cells). At the EAE onset, DCs from the CNS support inflammatory T cells at the peak of the disease, then become poor APC and prime the development of regulatory T cells (118–120). Notably, it was shown that DCs promote myelin-reactive T cells during the reactivation phase of EAE (121) leading to the proposition to target DCs for therapeutic approaches in MS. The accumulation of DCs during the neuroinflammation translating the EAE is becoming better understood. These immune cells can be mainly from peripheral bone marrow-derived precursors or from activation of resident microglia that will show DC characteristics, reflecting the wide range of DC precursors (120, 122). Another illustration is the role of DC during Guillain–Barré syndrome. They are the main APCs in the CSF, whether they are myeloid or plasmacytoid; they can present auto-antigen from spinal nerve, activate T and B cells, and finally lead to the autoimmune disease (123, 124).

Neuroinflammation can also be due to CNS injuries such as strokes or seizures. In these cases, different DC subpopulations can be recruited due to the release of various cytokines and chemokines by damaged cells. cDCs and pDCs have different sensitivities to chemokines according to their receptor expression (125). Studies have found peripheral derived DCs in the necrotic zone after stroke injury, but also resident DCs associated with T cells in the penumbral zone where they have a more immunomodulatory function after activation probably due to release of IFNγ (126). Others have shown the presence of DCs from bone marrow-derived population that exert pro-inflammatory actions with the production of cytokines after ischemic lesions (120, 127). Epilepsy is a consequence of infection or autoimmune disease, and during extended seizure, there are glial cell activation and upregulation of CAM expression on endothelial cells (128, 129). Also, in epileptic experimentation on rats, DCs can be found in blood vessels until 24 h after the seizure (130). In humans, the study of chronic epileptic patients has found the presence of DCs and T cells in blood vessels (131). In this epileptic context, DCs seems to maintain the chronic inflammation, source of seizures (132).

DCs are also important players during brain infections. During protozoal infection of the brain, caused by *Toxoplasma gondii*, DCs are major factors of neuroinflammation. First, the intracellular parasite accesses the brain through “Trojan horse” mechanisms using migratory leukocytes, including DCs (120, 133, 134). Next, during *T. gondii*-induced encephalitis, DCs found in the brain are mature APCs, produce pro-inflammatory interleukin (IL)-12 cytokines, and can provide Th1 response from CD4^+^ T cells (135, 136). Here, DCs participate in the initiation of immune response against the parasite, but they also maintain neuroinflammation and can contribute in *T. gondii*-induced encephalitis chronicity. For bacterial meningitis, the more severe CNS infection etiology, DCs are in high concentration in patient CSF. They lead to neuroinflammation by Th1 immune response induction, but they also exert a key role in the regulation of the host immune evasion of some bacterial strains such as *Escherichia coli* K1 (120, 137).

## Implication of Dendritic Cells in Viral Brain Infections

### Dendritic Cells as CNS Trojan Horses

DCs display also various roles in viral brain infections notably through their interaction with brain barriers. Indeed, especially mucosal and skin-resident DCs and LCs that are the first line of defense and an immune barrier against a multitude of external pathogens are also unexpectedly key actors of infection spread to the lymph nodes and other distal sites (8). Thereby, they can be viral carriers to the CNS by this inherent role as sentinel. These cells will also locally amplify the inflammatory response that, under certain conditions, can further aggravate infection outcome, and their activation can switch these protective cells to virus-transmitting cells (138, 139). However, there is currently a limited knowledge on the role of these cells in virus evasion, transmission, and systemic dissemination, especially when considering tissue-resident DCs and LCs upon flavivirus skin infection for instance, and their potential role in viral brain spread.

Concerning the recruitment mechanisms of DCs during viral infection, the release of chemokines or cellular adhesion molecules initiate the process. For instance, WNV- and Japanese encephalitis virus-infected neurons can produce CCL2 (140), and in EAE models, CCL2 released by astrocytes and endothelial cells will lead to chemoattraction of DCs (105). In this same model, immature DCs migrate in the CNS by interaction of α4β1 integrin with endothelial VCAM-1 and then participate in inflammation (106). Ou et al. (141) have also highlighted the role of VCAM-1 in DC recruitment during lymphocytic choriomeningitis virus infection in mice (**Figure 3**). Furthermore, an important expression and production of pro-inflammatory IL-1α and IL-1β have been reported during neuroinflammation in viral encephalitis (142, 143); IL-1 participates in lymphocyte activation and leukocyte infiltration by increasing CAM expression and other cytokine and chemokine induction. Also, IL-1 can be released by microglia and brain endothelial cells, leading to the enhancement of BBB damage and leukocyte recruitment (**Figure 3**). In that way, studies have demonstrated that during WNV infection, the IL-1 receptor (IL-1R1) participates in the activation of two types of DC populations with APC functions: the lymphoid-derived DCs and migratory DCs (144–146). And following WNV infection, *IL-1R1*-deficient mice have shown fulminant encephalitis (147), indicating a possible correlation between DC recruitment and neurological symptoms *via* IL-1 release. The involvement of BBB permeability during Japanese encephalitis virus infection in CD11^hi^DCs-ablated mice also illustrates the importance of these cells during regulation of brain infections (148). CXCL10 is another important chemokine for leukocyte recruitment in the brain. In HSV-1-infected mouse brains with CXCL10 deletion, there is a lower and even absence of NK cells, CD8^+^ T cells, and DCs, which translate to important viral loads with consequent CNS defects (149).

An important role of CD4^+^ T cells and DCs was also demonstrated in the dissemination of varicella zoster virus (VZV) at the BBB (40). Resident DCs and LCs from the skin and mucosa are the main carriers of VZV and VZV viral antigens to the lymph nodes where they can activate T cells. But they can also participate in viral dissemination through a “Trojan horse” mechanism (40). This mechanism has been reported in the case of other infections such as ZIKV brain infection (29) and WNV neuroinfection (20–22) or in a more older case of Maedi–Visna virus dissemination (150). For Nipah virus brain infection, reports establish that the principal access occurs through the transfer of infected DCs from the blood to the CNS (41). Toscana virus (TOSV), an arbovirus from the *Phlebovirus* genus, has been found in DCs from blood circulation of infected animals and humans. It was shown that TOSV-infected DCs notably produce IL-6 and tumor necrosis factor (TNF)-α, pro-inflammatory cytokines able to dysregulate the BBB. Thus, TOSV-infected DCs can be a pathway to reach the CNS (39). From the BCSFB side, it was shown that HIV-infected monocytes and DCs can allow viral invasion of the brain after CP passage. Thus, studies have proposed that HIV encephalitis emerges mainly by CNS infection through the choroid plexus (151, 152).

Numerous factors are implicated and are key regulators during the recruitment of DCs in the CNS, but the consequences of this recruitment must be discussed.

### Dendritic Cells and Brain Antiviral Response

Numbers of studies reveal the role of DCs in neuroinflammation during viral brain infections. DCs have major roles in antiviral responses, particularly through the regulation of T-cell responses. In a mouse model of viral encephalitis, the depletion of peripheral DCs led to a decrease of the viral presentation to CD8^+^ T cells and a delay in viral clearance from the brain, illustrated by an increasing death rate (103). Moreover, there is a complex role of DCs during HIV infection: they are at the entry site of the virus (mainly in mucosal areas) and can be infected and recruit CD4^+^ T cells, the main HIV reservoir (80). In the choroid plexus, they can also provide a CNS reservoir in HIV brain infection as mentioned earlier (153). During vesicular stomatitis virus infection, a model of acute encephalitis, DCs are the first responders at the olfactory bulb, the site of infection by this virus. Here, DCs activate and participate in the differentiation of T cells at early steps of neuronal infection (120). Similarly, in the Theiler’s murine encephalomyelitis virus-induced demyelinating disease mouse model, T cells are activated against endogenous myelin epitope due to an antigen presentation by DCs directly in the CNS (154). Epstein–Barr virus preferentially infects B cells and immortalizes them, but DCs play a role during this infection. Indeed, they can initiate innate and adaptive immune responses notably by viral antigen detection. Then, DCs can activate NK, CD4^+^, or CD8^+^ cells (155). However, a dysregulated CD8^+^ T-cell response against EBV can be at the origin of CNS impairments, and a link with MS development and recurrence is increasingly suggested (156, 157). From these last examples, DCs are clearly the activator of T-cell responses that will participate in viral clearance but consequently in neuroinflammation.

In response to viral infection and because they express high TLR7, TLR9, and interferon regulatory factor7 (IRF7), pDCs are the main IFN type I producers (158). They recognize PAMPs as viral antigens through their PRRs leading to a production of type I IFN and transcription of ISGs. For instance, when DENV particles or DENV-infected cells are brought into contact with peripheral blood mononuclear cells (PBMCs), the first response is a secretion of IFNα by pDCs (159). It has been shown that direct DC infection by the neurotropic arbovirus Usutu virus (USUV) induces strong IFNα production and, in comparison to WNV-infected DCs, USUV seems to be more sensitive to this type I IFN response. This can partly explain that the percentage of neurological clinical disorder in human following WNV infection is higher than USUV infection (160). Nonetheless, during WNV infection, DCs are mostly found in brain tissue of mouse models where there is a predominance of *IFNα* mRNA (161).

As already noted, DCs have an important function during epileptic seizure, and some viral infection of the CNS can lead to epileptic syndrome such as SARS-CoV-2 infection (162), arbovirus infection (30, 163–165) or during HIV infection and HSV encephalitis. Incidentally, studies reveal that a large proportion of epileptic seizures could be from infectious etiology and that a lot of work needs to be done in that domain (166). Notably, during epilepsy studies on guinea pig brains, the release of neuroinflammatory factors was described at the BBB (129). These are selectins and CAMs, known to attract leukocytes as DCs. In this context, it appears that DCs could be an interesting target for therapy (167).

Finally, various DC phenotypes are recruited depending on the virus and host immune responses. *In vivo*, the mouse hepatitis virus model shows two DC populations of which the most predominant expresses co-stimulatory molecules (CD40, CD80, and CD86). This DC population is responsible for effector CD8^+^ T-cell activation upon CCL3 modulation (168). The two distinct pDC and cDC populations can be detected in the brain of HSV-1-infected mice, and it was shown that respective CCR knockout models have increased susceptibility to the virus, indicating a complementary role (149). In a cohort study of coronavirus disease 2019 (COVID-19) patients with neurological impairments, it appears that DCs found in the CSF have a different profile compared to the normal DC subset found in the CSF of healthy controls. They are more susceptible to interaction with CD8 and CD4 T cells and have a critical role during the emergence of neuronal symptoms (169). More generally, each DC subtype can be recruited during CNS infection following specific neuroinflammatory mediators (170).

In these examples, DCs are important to control virus spread in the systemic circulation, but they are also transporters of virus to the CNS or promoters of neuroinflammation. In addition, several DC subpopulations can have a role during the same infection. Hence, the importance of their study to boost antiviral response and control virus spread and neuroinflammation that can have serious and long-term consequences.

## Discussion

In conclusion, it appears that 1) viral brain infection can be mediated by several mechanisms and lead to neuroinflammation with various consequences including leukocyte recruitment; 2) as a result, immune cells and particularly DCs can interact with brain barriers and invade the CNS to act against the viral infection; 3) they can also support neuroinflammation; 4) and they can be a “Trojan horse” during virus CNS entry. Indeed, a viral infection can lead to general inflammation as seen with a cytokine storm that can have a deleterious effect on the CNS with chronic neuroinflammation leading to various sequelae (171, 172). This inflammation, as well as by DC innate properties, can lead to recruitment of DCs in the CNS (125), sometimes also carrying the virus itself in the brain (40, 72). Once in the CNS, DCs can directly act against viruses, for example, with IFN production (173), they also contribute to neuroinflammation by T-cell activation for instance (170). For each of these conditions, different DC populations can be recruited (120). However, to tackle these pathological manifestations, we need a better characterization of CNS inflammation following infections. Thus, there is a major need to better dissect the role of each cell type in the establishment of neuroinflammation processes by considering both neuronal and immune cells including DCs. Novel technologies allowing quantitative single-cell mRNA sequencing and proteomic profiling of inflammatory markers may reveal specific DC characteristics linking these cells with the establishment of inflammation and CNS disorders. These data will critically contribute to deepen our current knowledge on brain viral infection. Moreover, DCs are known for their role in priming T-cell responses. It will be, therefore, of interest to further characterize how DC-dependent antiviral T responses can lead to neuroinflammatory pathologies and what are the antigenic drivers of such conditions. Clarifying the role of DCs as major players in neuroinflammation as well as deeper investigations on how they are carrying viruses to distal organs will undoubtedly render the possibility to consider them as a therapeutic target in order to rapidly control the possible pathological outcomes of infections. Gathering more data on metabolic and immunobiological features of DC subsets at sites of primary viral infection will also allow to transpose and adapt novel antiviral therapies, for example, by a topical drug-based application targeting early events of skin DC infection.

Moreover, a flavonoid, apigenin, was proposed to regulate inflammation by a reduction of α4-chain expression by DC and limiting the BBB cross (174). In the context of MS, there are some studies as the use of prestimulation of DC TLR9 to increase neuroinflammation control and immune regulation of the course of the disease in EAE (175). The use of DC-vaccine was also proposed to support the treatment of neurodegenerative disease as Alzheimer’s disease (176). Nonetheless, in the context of viral brain infection, there is a lack of evidence for the use of DC in care. Also, the regulation or the blocking of DC entry into the CNS should be explored, for example, to limit viral carrying. Increasing work on different subsets of DCs depending on the viral infection etiology could be a key issue to understand and treat neuroinfections that are considerable public health concerns.

## Author Contributions

SS conceived the study. OC, GM, FB, YS, and SS wrote the review. PVD corrected and amended the article. All authors contributed to the article and approved the submitted version.

## Funding

This work was publicly funded through ANR (the French National Research Agency) under the “Investissements d’avenir” program with the reference ANR-16-IDEX-0006 » and by la Région Occitanie through the PhD funding program with the reference R19068FF.

## Conflict of Interest

The authors declare that the research was conducted in the absence of any commercial or financial relationships that could be construed as a potential conflict of interest.

## Publisher’s Note

All claims expressed in this article are solely those of the authors and do not necessarily represent those of their affiliated organizations, or those of the publisher, the editors and the reviewers. Any product that may be evaluated in this article, or claim that may be made by its manufacturer, is not guaranteed or endorsed by the publisher.
